# Strategies to Engage Blacks in Sleep Medicine: Lessons Learned from Three Studies Applying Community-Based Participatory Research Principles

**Published:** 2023-05-26

**Authors:** April Rogers, Alicia Chung, Azizi Seixas, Debbie Chung, Ferdinand Zizi, Girardin Jean-Louis

**Affiliations:** 1 Department of Health and Human Services, St. John’s University Collins College of Professional Studies, Queens, New York, 11432,USA; 2 Department of Population Health, Center for Early Childhood Health and Development, Division of Health and Behavior, New York University Medical Center, New York, NY 10016, USA; 3 Department of Informatics and Health Data Science. Center for Translational Sleep and Circadian Sciences, University of Miami Miller School of Medicine, Miami, FL, 33136,USA; 4 Department of Psychiatry and Behavioral Sciences, Translational Sleep and Circadian Sciences (TSCS), Miami, FL 33136, USA

**Keywords:** Sleep, Health disparities, Community engagement, Obstructive Sleep Apnea (OSA)

## Abstract

**Introduction:**

Awareness, assessment and treatment of sleep apnea are disproportionately lower among Blacks, compared to other racial/ethnic groups. To address this health disparity gap, communication strategies that connect Blacks to OSA education, detection and treatment adherence interventions are needed. Strategies that engage individuals through communication technologies, community-level social network support, and medical providers in clinical settings are also needed. We present lessons learned from three studies that offer these solutions using a community-engaged research model: The Metabolic Syndrome Outcome Study (MetSO), Peer-enhanced Education to Reduce Sleep Ethnic Disparities (PEERS-ED), and Tailored Approach to Sleep Health Education (TASHE), to capture program effectiveness and lessons learned from project successes and failures.

**Methods:**

The methods of OSA community-based programs included the application of an OSA community-engaged research model. This model served as a strategic guideline for effective interventions to engage communities in research and ensure cultural appropriateness in OSA interventions. Focus groups, in-depth interviews and community steering committee meetings were conducted with various stakeholders. Delphi surveys were used to identify high priority diseases and conditions. Community barriers and needs were identified through iterative combinations of surveys and focus group meetings. Stakeholder groups participated in all aspects of our studies, including the development, dissemination and implementation phases, reflecting a bi-directional decision-making process that ensures the interests of both parties were considered. The MetSO, PEERS-ED and TASHE studies were reviewed to understand the effectiveness of the programs and to evaluate lessons learned.

**Results:**

MetSO, PEERS-ED and TASHE interventions revealed that community-engaged strategies are successful in enrolling Black populations into clinical trials. The study teams reached nearly 3,000 Blacks at risk of OSA and screened about 2,000 people in sleep apnea studies in New York City. Sleep brochures were distributed to over 10,000 people. Lessons learned from MetSO, PEERS-ED and TASHE interventions revealed that building relationships, establishing trust, identifying a study champion, learning to adjust and providing participant incentives are key strategic elements for successful recruitment and retention of Blacks participations in clinical trials.

**Conclusion:**

Strategic application of community-oriented frameworks ensures active community engagement throughout the research process, allowing for greater enrollment of Blacks in clinical studies as well as increased OSA awareness, diagnosis, and treatment.

## INTRODUCTION

Increasing the participation of Black populations in clinical trials has frequently been identified as a highly significant goal for health Scientist [1], and compliments the foundational principles of Healthy People 2030; of which, notes that achieving health and well-being is a shared responsibility towards efforts to eliminating health disparities and increasing health equity [2]. In addition, Black populations have historically shouldered a disproportionate burden of adverse health outcomes, with higher prevalence of obesity, cardiovascular disease as a sleep disorders, primarily influenced by a higher exposure to social determinants of health, which are shaped by social, economic and political factors [3]. The participation of Blacks in clinical trials is vital to understand the health needs of a community, to improve scientific robustness and to overall improve health equity [4]. Obstructive Sleep Apnea (OSA) is defined as sleep breathing disorder associated with recurrent apnea and hypopnea episodes due to complete or partial collapse of the upper airway [5]. In the United States, Blacks are disproportionately affected by OSA [6]. Strategies to engage and provide Blacks with OSA educational materials, information about OSA detection and treatment options in clinical settings are currently limited [7]. Barriers for enrollment of Blacks into clinical studies have been challenging for both personal reasons (i.e., lack of trust in scientists, once data is collected participant is “forgotten”) and pragmatic (i.e., lack of transportation, inability to take time off from work) [1]. Other barriers to engage in clinical trials can include participant poor health literacy and insufficient support navigating the healthcare systems [1]. The influence of culture on beliefs and attitudes may play a major role in shaping how health communication messages and evidence-based interventions are perceived among Black populations [8]. A person’s understanding of a health condition may influence perception of risk, which is built through exposure to baseline knowledge, media coverage, and learned or observed information from family and friends [9]. Furthermore, evidence suggests that a combination of beliefs, risk perception and attitudes may contribute to lifestyle behaviors that are associated with risk factors for chronic diseases including diabetes, metabolic syndrome and OSA [9]. Strategic approaches that a bridge beliefs, knowledge and attitudes are needed for effective communication with Blacks to increase uptake of health information.

This paper describes the lessons learned while implementing community-engaged approaches in three studies Metabolic Syndrome Outcome Study (MetSO), Peer-Based Sleep Health Education and Social Support (PEERS-ED) and Tailored Approach to Sleep Health Education (TASHE), conducted among Blacks. Our experiences from conducting the three clinical trials may provide important insights into successful strategies to enhance participation of Black populations in interventions to improve sleep health.

## MATERIALS AND METHODS

An independent program evaluator conducted an evaluation of MetSO, PEERS-ED and TASHE clinical research studies. The evaluation process was guided by “A Guide to the Project Management Body of Knowledge” (PMBOK^®^Guide) [10]. The protocols for individual research projects (MetSO, PEERS-ED and TASHE) were reviewed and assessed by the research team and program evaluator. MetSO, utilized a physician-patient engagement approach at community clinical settings, with physicians providing health advice and knowledge regarding OSA. In PEERS-ED, patient-peer health champions, who learned and experienced issues related to OSA themselves, were the primary source for health information for participants [7]. In TASHE, participants received culturally tailored health advice, knowledge and testimonials from individuals who experienced OSA health related issues, in addition to published health information provided on electronic devices (i.e., iPads)[11]. In MetSO, Peers-Ed and TASHE, engagement among physicians, researchers, health champions and community stakeholder were garnered by prior relationship building with the community (i.e., churches, barbershops, salons, and community centers) that were fostered over years of mutual trust, transparent communication, and a mutual understanding of community needs. Table 1 provides a summary of the research studies objectives and shared interventions.

## RESULTS

### Recruitment outcomes (MetSO)

The Metabolic Syndrome Outcome Study (MeSO) funded by the National Institute of Health (NIH) is a randomized control trial that examined the efficacy of a culturally and linguistically tailored behavioral telephone-delivered intervention to increase adherence to physician-recommended assessment and treatment of OSA. The MetSO theoretical framework is guided by the transtheoretical model developed by Prochaska, which focuses on the phenomena of intentional change [12]. Utilizing the transtheoretical model MetSO sought to examine the relationship between participants readiness to use Continuous Positive Airway Pressure (CPAP) and success to achieve increase adherence to physician-recommended OSA and CPAP treatment. MetSO recruited 1035 Blacks with Metabolic syndrome in a primary-care setting in four clinics associated with SUNY Downstate Medical Center in Brooklyn, NY [5]. Participants were randomly assigned to either a Tailored-Telephone Intervention (TTI) arm or an control arm. The intervention content was delivered by a trained health champion who had experience working with minority populations. The intervention arm content provided culturally and linguistically tailored information that took into consideration the unique culture and ethnic norms and values of the target population. Before the implementation of the TTI, focus groups with the target population was conducted of which, telephone tailored intervention information was tested. Study investigators relied on a community advisory board to provide input regarding the intervention design and content, providing an opportunity for appropriate messaging. Participants in the control arm received standard information about obstructive sleep apnea from the National Institute of Health and the American Academy of Sleep Medicine. All participants from the registry were selected to estimate risk of sleep apnea using the Apnea Risk Evaluation System (ARES^™^) Questionnaire. Questionnaires were administered in a primary healthcare facility by trained healthcare educators. Questions on sleep patterns, daytime functioning, and knowledge of sleep apnea were also assessed. Of the 1035 participants who completed the ARES questionnaire, 48% were at high risk for OSA [6].

### Key best practices followed and lessons learned

Key best practices followed and lessons during MetSO were as follows:

- The intervention was culturally and linguistically tailored to address the needs of black participants. We believe that interventions should take into consideration the unique cultural and ethnic norms and values of the target population in order to have a greater likelihood of success.
- It was based on evidence that telephone-delivered interventions are effective for increasing rates of screening and treatment for various health problems, including colorectal cancer and depression, appointment keeping, and adherence to treatment.
- Telephone-delivered interventions are cost-effective and participants do not need to leave their home.
- Application of the transtheoretical model, allowed each phone contact to be individually tailored to assess stage of readiness as well as the participant’s level of motivation.
- Exit interviews with all the participants permitted the participants to complete the same baseline questionnaires and additional questionnaires designed to understand why participants adhered to the intervention and how the intervention might be refined to be more effective.
- An internal data and safety monitoring committee was developed, in compliance with NIH regulations. The purpose of the committee was to ensure the safety of participants and the validity and integrity of the data.
- A comprehensive plan for training the Health Champions (HC) was essential to the development of the intervention. HC received 30 hours of training in sleep medicine, and will be continuously monitored throughout the study period to ensure adherence to study protocol and ascertain knowledge and application of the AASM obstructive sleep apnea assessment and treatment guidelines.
- Materials were piloted and tested before implementation during conducted formative research, including focus groups with the target population.
- Community advisory board provided input on the intervention design, including program materials for specific content areas, such as appropriate areas, such as appropriate messaging and education levels.
- The program Health Champions worked with patients who missed their appointments until they were rescheduled.
- Once the appointments were rescheduled, patients received a positive reinforcing message where they were congratulated and thanked.
- Patients randomized to the TTI condition received up to 10 phone calls during the 6-month trial. Consistent with previous research an average of 5 calls, lasting up to 25 mins each was necessary to achieve desired outcomes.
- The most effective, noninvasive treatment for sleep apnea requires the use of Continuous Positive Airway Pressure (CPAP).
- The integration of qualitative research with the randomized controlled trial design is unique because the four focus groups (two with each arm) with a sample of participants 6 months after completion helped to ascertain what the participants liked and disliked about the intervention.
- Repeated-measures MANCOVA used to examine differences between baseline and follow-up measures. MANCOVA is a robust technique that incorporates routines to account for heterogeneous variances, serial correlations and variance inflation.
- The program Health Champions endeavored to enhance patient’s comfort and confidence using PAP until the end of the 6-month period when outcome ascertainment occurred.
- In cases where resistance to treatment was encountered, the Health Champions provided further education on the benefits on PAP therapy.
- By implementing an intervention tailored to address specific challenges Black patients face, as well as delivering the messages according to patient’s stage of readiness, we were able to demonstrate a significant uptake of tailored OSA messages leading to desired behavior change.

### Recruitment outcomes (PEERS-ED)

PEERS-ED is a randomized controlled trial funded by the NIH to address barriers among Blacks through a culturally tailored peer education program based on motivational enhancement principles. The study’s theoretical framework is based on the following four elements from the Centers for Disease Control AIDS Community Demonstration Projects: 1. Community assessment to determine project aims; 2. Socially desirable risk reduction; 3. Mobilization of credible peers to dispense sleep health education; and 4. Role modeling to disseminate health messaging [13,14]. Community engagement served as a vital component to the structural foundation of this study’s development, as indicated by the first element of the theoretical framework. The lead health educator, who embodied community activism and strong interpersonal skills, identified, empowered and trained Community Steering Committee (CSC) members. Community Steering Committee members served as the critical link for bridging research and practice, by engendering trust with participants and a relationship with community leaders. The CSC guided the study’s development, and community health educators were a central component to the intervention. Community recruitment involved engaging barbershops, nail salons, community-based-organizations and houses of worship. Upon initial screening, the two-arm study randomly assigned people to either the tailored peer education intervention arm or standard educational brochures in the control arm over a 12-month.

Participants in both arms were enrolled in a home-based study, including OSA evaluation with WatchPat. Participants with Apnea Hypo-Apnea Index (AHI) ≥ 10 were referred for sleep diagnostic evaluation at a clinic. Participants diagnosed with OSA were provided a Continuous Positive Airway Pressure device, and were encouraged to adhere to the recommended treatment through a racially and ethnically tailored, peer-based education program. Measures collected at baseline, 6-month and 12-month follow-up include socio-demographic variables, the Epworth sleepiness scale, the Apnea Knowledge Test and Apnea Beliefs Scale, the change assessment scale, medical history, and the Apnea-Risk Evaluation System questionnaire. Both groups committed to follow-up outcome assessment. Recruitment for this study began in 2015. 319 people were enrolled in the intervention and control arm. Participant feedback during study debriefs indicated that a web-based educational source on obstructive sleep apnea would allow for continuous access and fill the void between in-person sessions with their health educator [13]. Given the pervasiveness of online devices and internet connectivity among racial and ethnic minority communities, a tailored web-based sleep health educational tool was warranted. To this end, our third community engagement informed study was an online educational resource named TASHE.

### Key best practices followed and lessons Learned

Key best practices followed during the PEERS-ED study were as follows:

- Peer health education has emerged as a successful model integrating a caring and trust-building approach leveraging credible health champions sanctioned by community leaders.
- Peer educators must in turn be conversant in a specific health area and intimately familiar with social sanctions, rituals, values, and rules of conduct within the community to ensure intervention goals are achieved in order to gain trust from the intended audience.
- Peer education is an effective tool to motivate Blacks with OSA symptoms to engage in healthful practices in line with their own level of readiness and efficacy to navigate the healthcare system
- The role of interpersonal influence, a fundamental component of peer health champions, has a profound effect on anchoring behavioral change.
- Direct engagement is a very effective approach in addressing OSA barriers.
- Participants assigned to peer sleep health educators received additional information regarding the lab-based procedures and further support as participants navigated this phase of their OSA care; they also received assistance with scheduling appointments as the need arose.
- Blacks revealed that receiving social support from family and/or friends had a greater usage of OSA treatment.
- Higher level of social support is generally associated with greater adherence to medical care among patients with chronic health conditions.
- It is well established that patients with OSA experience clinically meaningful improvement in metabolic conditions.

### Recruitment outcomes (TASHE)

TASHE, a two-arm randomized controlled trial developed a culturally and linguistically tailored website that provided information and interactive vignettes as a platform to ascertain whether tailored materials would increase OSA knowledge and health literacy by comparing Blacks exposed to tailored materials versus those exposed to standard sleep health literature [14]. This study was funded by the National Institute of Health (NIH). TASHE utilized a community-engagement approach, recruiting participants from places of worship, barber shops, beauty salons and community organizations. A total of 524 potentially eligible individuals were contacted and recruited, 194 of whom met eligibility criteria and were enrolled into the study. Eligibility criteria included those who self-identified as black, African American, or Caribbean American; were 18 years of age or older; and were at moderate or greater risk of OSA, determined as a score of 4 or greater on the Apnea Risk Evaluation System. The study utilized iPads to disburse OSA literature and tailored information to participants [15]. In this approach, the study sought to dismantle negative beliefs and attitudes about sleep and OSA and increase educational information about health literacy. In our attempt to dismantle negative beliefs, patient testimonial was captured on video, which allowed for an intimate interaction, flexibility in viewing times and the ability to re-watch materials. Through testimonials, participants were able to watch video testimonials of Black participants who had undergone sleep studies, learning from their personal journey and experience with OSA. It is through such a poignant interaction that participants start to feel safe, and truly develop an understanding of OSA. Patient testimonial videos, educational infographics, and OSA educational content were delivered on an iPad that only had access to a website content at a literacy level appropriate for the targeted audience. The intervention revealed TASHE exposure significantly increased OSA self-efficacy (OSA outcome expectation [β =0.5; 95% CI: 0.1-0.9] and OSA treatment efficacy [β=0.4; 95% CI: 0.0-0.8]) at 2 months but not at 6 months. Additionally, TASHE exposure improved sleep hygiene at 6 months (β=6.7; 95% CI: 2.2-11.3) but not at 2 months.

### Key best practices followed and lessons

Key best practices conducted during TASHE included the following:

- Using themes from focus groups to develop a culturally and linguistically tailored website and two vignettes.
- Use of the Taylor model as the framework guiding role model story development.
- Pretesting all role model stories and vignettes before randomization.
- Two-arm randomized controlled trial inclusive of a culturally and linguistically tailored website.
- Bringing together academic investigators, community providers, and stakeholders. This is considered to be an effective tool to develop interventions, sustainable programs, and policies to address health disparities, and to improve adoption of healthful practices in underserved communities.
- Use of the partnership approach that combines knowledge with action to achieve social change in order to improve health outcomes and eliminate health disparities.
- Convening a Faculty Advisory Board (FAB) comprised of the leading sleep medicine physicians, scientists, and researchers to help develop (review and approve) sleep health content of all TASHE materials before dissemination.
- The Community Steering Committee (CSC) that brought together community stakeholders, patients, and health advocates working with the TASHE project leadership and FAB.
- Use of Google Analytics to track website usage and traffic.
- Recruitment of potential role models/influencers from the community to be screened.

## DISCUSSION

Results of the MetSO, PEERSED and TASHE interventions show that community-engaged strategies are successful in enrolling Black populations into clinical trials. The ill-defined reference to Black populations as ‘hard-to-reach’ is misguided and reflects a lack of regard for available scientific data and inadequate research engagement efforts, resulting in failure to reach minoritized communities [16]. The MetSO, PEERS-ED and TASHE trials have been successful in meeting all study goals, providing confidence that future studies using similar approaches may yield similar outcomes.

### Recommendations for recruitment and retention

Based on the literature and our findings regarding research studies MetSO, PEERS-ED and TASHE, we offer the following recommendations for in-person recruitment of Black populations for research studies.

#### Relationships:

Relationships between Community Steering Committee (CSC), health educators, small business owners, community organizers and pastors of faith-based organizations stem from respect, shared vision and existing and continuing rapport. SUNY Downstate Medical Center in Brooklyn, New York was a recruitment site for MetSO, PEERS-ED and TASHE [16]. The relationship SUNY Downstate Medical Center fostered with participants, health educators, researchers, physicians and other project stakeholder provided a strong platform built on trust that aided in the success of the studies. A Partnership with The Arthur Ashe Institute for Urban Health, located in Brooklyn, collaborated with community members to design and implement interventions that address health conditions that disproportionately affect minority populations [17]. The linkage between SUNY Downstate Medical Center and organizations such as the Arthur Ashe Institute for Urban Health allowed for mutual visions among elected officials, community leaders, local business, and community based organizations and individuals in the community to participate in creating solutions to health concerns.

#### Trust:

Historical injustices with research in communities of color have driven mistrust of academic institutions. Patient mistrust, perceived racial discrimination, transparency, culture, language, health literacy, lack of social support, and pre-existing co-morbidities all contribute to racial and ethnic minority barriers of trusting academic institutions to engage in research [18-24]. Without trust, community-based research cannot be effective. Trust allows for strong partnership development and capacity building. During the five-year execution of the projects, staff and personnel rotation were expected. To minimize any disruption that staff and personal rotation would bring, it was important for the projects to maintain a trust-worthy reputation within the community. Trust can be built by ensuring a firm execution plan with detailed protocols. In building trust within the community, it is also important for key personnel to maintain a visible presence. During the MetSO study, research staff maintained a five day a week presence at the recruitment site. Physicians assisting with recruitment were in regular communication with research volunteer of whom aided with providing questionnaires and to answer questions about the study protocol. In the TASHE and Peers-Ed studies, research staff maintained weekly communications with community leaders at recruitment sites *via* phone, email or text. Investing time to prepare project stakeholder to engage and achieve project goals strengthens relationships and shows commitment. During the implementation of MetSO, PEERSED and TASHE interventions, participants were more inclined to engage in the study if they were able to feel a sense of trust among those who were in charge of providing health related information. The three research studies required the participation of individuals in the community, indicating the critical role of trust in the ability to recruit participants to the studies.

#### Study champion:

Identify a “study champion” to facilitate communication between research participants, staff and community stakeholder. The MetSO, PEERSED and TASHE trials benefited from identifying and supporting a study champion. A study champion is someone who is familiar with the research study, friendly with the research staff and clinical staff and a encourager, an advocate for the research, community and participant [4].

#### Learn to adjust:

Participants and other community stakeholders may experience other demands of life that may interrupt the natural progression of recruitment.

#### Provide participant incentives:

Participants may be more inclined to engage in research if there is a monetary incentive involved. Furthermore, we need to acknowledge that incentives may not always be monetary. In our research studies, some participants were looking for long-term engagement to not become a 'forgotten participant'. Remaining in contact with participants have been one of our greatest strategies for recruiting, retaining and engaging for research studies.

## CONCLUSION

To increase health equity and dismantle the old rhetoric of Blacks regarded as a 'hard-to-reach' population, effective strategies must be utilized to effectively increase recruitment success of Blacks in research studies. We have learned that trust building and shared goals and values are earned over time. It is essential for scientists to approach recruitment and retention in a way where future scientists can build upon and expand existing relationships.

## Figures and Tables

**Table 1:** Summary of program objectives and shared interventions.

Interventions								
Program objectives	Sponsored program	Education	TTI	PHE	Online platform	Materials dissemination	Community partners	Cultural linguistic tailoring
Increasing health literacy	TASHE PEERS-ED	√	√	√	√	√	√	√
Increasing access to information	TASHE PEERS-ED MetSO	√	√	√	√	√	√	√
Contextualizing informational materials through uses of different delivery formats to remove barriers	TASHE PEERS-ED MetSO						√	√
Increasing adherence to recommended sleep consultation	PEERS-ED MetSO	√	√	√	√	√	√	√
Increasing adherence to treatment recommendations	PEERS-ED MetSO	√	√	√	√	√	√	√
Bridging the gap between communities and healthcare system	PEERS-ED MetSO	√	√	√	√	√	√	√
Assisting in navigating the healthcare system	PEERS-ED MetSO	√	√	√	√	√	√	√
Advocating for individual and community needs	PEERS-ED MetSO	√		√		√	√	√
Building individual and community capacity	PEERS-ED MetSO	√		√		√	√	√

**Note:** TTI=Tailored Telephone Delivery Intervention; PHE=Peer Health Educators.
